# Mortality: life and health expectancy of Canadian women

**DOI:** 10.1186/1472-6874-4-S1-S9

**Published:** 2004-08-25

**Authors:** Marie DesMeules, Douglas Manuel, Robert Cho

**Affiliations:** 1 Centre for Chronic Disease Prevention and Control, Health Canada, 120 Colonnade Rd, Ottawa, Canada; 2 Institute for Clinical Evaluative Sciences, 119, 2075 Bayview Ave, Toronto, Canada; 3 Centre for Chronic Disease Prevention and Control, Health Canada, 120 Colonnade Rd, Ottawa, Canada

## Abstract

**Health Issue:**

The sex differences in mortality, life expectancy, and, to a lesser extent, health expectancy, are well recognized in Canada and internationally. However, the factors explaining these differences between women and men are not well understood. This chapter explores the contribution of various causes of death (such as preventable, and sex-specific deaths) on these differences between women and men.

**Key Findings:**

"External" preventable causes of death (e.g. smoking-related, injuries, etc.) were responsible for a large portion of the sex gap in mortality and life expectancy. When excluding these causes from the calculations, the sex gap in life expectancies were largely reduced, decreasing from approximately 5.5 years (life expectancy being 81.4, years in women, and 75.9 years in men) to approximately 2.2 years (84.9 in women and 82.7 in men). Sex gaps in corresponding health expectancies entirely disappeared when these preventable causes of death were excluded. Moreover, a larger death burden was observed among women than men for sex-specific causes of death (eg. excess breast cancer, gynaecological cancers, maternal mortality). Significant disparities were also observed in the mortality rates of various subgroups of women by geographic regions of Canada.

**Data Gaps and Recommendations:**

These results indicate that women do not appear to have a large biological survival advantage but, rather, are at lower risk of preventable deaths. They also provide additional information needed for the development of policies aimed at reducing disparities in life and health expectancies in Canada and other developed countries.

## Background

Mortality and life expectancy are well-established and commonly used indicators of population health, and important patterns by sex and over time have been observed in Canada [1] and internationally. [2] Canadians' life expectancy, estimated at approximately 76 years for men and 81 years for women, [1] has consistently ranked among the top 10 of all OECD (Organization for Economic Co-operation and Development) countries for several decades. [1] The relative difference in mortality and life expectancy between men and women is well documented in the existing literature. [1-4]

The overall decrease in mortality and the corresponding increase in life expectancy over the last century among men and women are also well known. Women who were born at the beginning of the twentieth century could expect to live to about 50 years of age, three years longer than men. This gap in life expectancy reached a peak in the early 1980s (over seven years' difference), has been narrowing since then, and is currently approximately five years. [3] Although the mortality rates of Canadian men are currently higher across all age groups, this was not the case historically. For example, until the 1940s, mortality rates among Canadian women aged 20 to 49 were actually higher than those among men in the same age group. [5] These important gains in life expectancy have been explained by a number of factors, including a reduction in infant mortality and maternal deaths. In particular, approximately half of the increase in life expectancy before the 1980s can be attributed to a decline in infant mortality, whereas more recent increases are due to a decline in the mortality rate of older people. [3] Over the latter half of the twentieth century, a 52% reduction in the female age-adjusted mortality rate from all causes combined has been observed, as compared with a 39% decrease for males. However, from 1990 to 1997, the death rate declined by twice as much among males as it did among females. [1] Patterns by cause of death also vary between women and men, especially in adulthood (age 20 to 64). [6]

Canadian women have a longer health-adjusted life expectancy than men, [7] although the sex gap is less pronounced than that of life expectancy. This is in part explained by the higher prevalence of a number of disabling chronic conditions among Canadian women (see the Second Report on the Health of Canadians); [21] therefore, health expectancy is another useful, gender-sensitive indicator of women's health. As infectious diseases have declined as a major cause of death over the past century, people now survive to older ages, when susceptibility to chronic diseases is greater and functional limitations are more common. Therefore, this indicator has gained significant recognition in population health in recent years. [7]

Even though Canadians, and women in particular, have a relatively long life expectancy overall, important disparities exist. Regional variations have been observed within Canada, including provincial/territorial as well as north-south differences in life expectancy. Provincially, there is an east-west gradient in life expectancy, with the shortest life expectancies observed in Newfoundland and Labrador and the longest in British Columbia. [3] Although life expectancies have been increasing in the Territories since the 1950s, they still remain lower than in other parts of the country. [4]

Socio-economic factors such as income and education also have a significant impact on life expectancy. [8,9] Moreover, differences in health expectancy by socio-economic status can exceed differences in total life expectancy. [8] These disparities are also relevant to gender, since the greatest differences in life expectancy between women and men were seen in the most deprived areas. [8,10] The impact of income on mortality and the difference in mortality between the geographic areas with the highest and the lowest income levels have been found to be smaller for women than for men in Canada and other developed countries. [8,10] It is unclear whether this less pronounced effect among women may be due to the less gender-sensitive measures of income used (neighborhood income rather than individual income). Most studies to date on socio-economic status (SES) have used an ecological approach, which has recognized limitations and which may produce less reliable risk estimates, especially for women. These disparities in mortality by income have decreased in absolute, if not relative, risk terms over the last decades in many countries, including Canada. [8,10]

The Canadian population is very diverse in terms of ethnicity and culture. Although there is a paucity of information on mortality patterns by ethnicity in Canada, it is well established that First Nations people in general have poorer health and higher mortality than non-Aboriginal Canadians. [11-13] This has also been observed among Native populations in other countries. [13-15] Sex and gender differences with respect to these disparities among First Nations people have not been well described.

Health-related behaviours such as smoking and physical activity also have obvious impacts on mortality and life expectancy. [16-19] Given that patterns of SES and health behaviours differ significantly between women and men, they are particularly relevant in the context of this report, and more detailed discussions of health-related behaviours, socio-economic factors and ethnic diversity are included in other chapters of this report.

Biological and hormonal factors, such as age at menopause, also play a role in women's risk of mortality. Mortality risk has been found to be somewhat increased among women who have had natural or surgical menopause before the age of 46. [20] The highest relative risk was found among women with the highest age at last birth, but there is controversy regarding the effects of late pregnancy on mortality risk. [20] Other determinants of mortality, such as psychosocial and environmental factors, have also been described in the literature as having an impact on mortality and are established determinants of health. [21]

Although mortality and life expectancy have been well described in the Canadian population, there is a paucity of information concerning the sex and gender gaps in mortality and life expectancy, in particular the factors contributing to the sex gaps observed, and how gender-relevant determinants affect the mortality of subgroups of Canadian women. Since many factors affecting mortality (such as the effect of hormonal, psychological or environmental factors) are beyond the scope of this chapter, key descriptive population health measures have been selected and are presented here for the purpose of women's health surveillance. To explore these sex and gender differences further and provide additional insight, preventable mortality and its effects on life expectancy are examined. Similarly, biologically based differences in mortality and life expectancy are presented. Various measures of health expectancy, a useful indicator of women's health, are also examined more comprehensively. Finally, mortality patterns in subgroups of women are presented.

## Methods

Two main national data sources were used for the analyses presented here. Mortality rates and life expectancy were calculated using annual Canadian mortality data (1959–1999) and census data. General mortality patterns and age-adjusted mortality rates over the last four decades are described by major causes and sex. Sex ratios (male over female mortality rates) are also presented.

To identify the relative contribution of various preventable and non-preventable causes of death to the sex gap, causes were classified into the following categories according to established criteria: preventable/avoidable through primary prevention (e.g. smoking-related, accidents, etc.), [22] avoidable through medical intervention (amenable causes such as asthma), [23] sex/biologically based (e.g. female cancers, prostate cancer, etc.) and other (see Additional data file at the end of this chapter for a more detailed description). The relative contribution of the first three broad categories of conditions to the sex gap in mortality and life expectancy was estimated by calculating "cause-deleted" mortality and life expectancies. [The authors recognize that the "complete" prevention scenario, in which 0% of the population would be non-smokers and 100% of accidents would be prevented, is not realistic from a public health perspective. However, these artificial hypotheses are useful in isolating the relative contribution of preventable and non-preventable causes to sex and gender gaps in health and in determining the maximum potential impact of possible interventions on reducing these sex and gender gaps in health.] These three categories are not mutually exclusive, as some diagnostic categories are included in two categories (for example, a proportion of heart disease mortality is preventable through primary prevention and is also preventable through appropriate medical intervention).

Cause-deleted mortality rates were calculated by subtracting the age-specific mortality rate from selected causes from the overall mortality rate for each age-sex group. An age-standardized rate was then calculated using the 1991 Canadian population as standard. Potential years of life lost (PYLL) to age 75 and the associated age-standardized rate were also calculated. Life table analyses were used for the calculation of life expectancy. [24] Health-adjusted life expectancies (HALEs) were calculated using a measure of health-related quality of life (derived from the Health Utilities Index [25]) from the Canadian Community Health Survey (2000). This index, HUI3, classifies respondents into physical functioning levels ranging from unrestricted to a highly disabled state. [26] HALEs were then calculated by applying various weights to years of life, based on the level of disability. [27]

Comparisons of mortality rates between rural and urban women were made by categorizing census subdivision (CSD) of residence for all death records (1986–1996) into five categories, from "rural" to "urban" using a classification method developed by Statistics Canada. [28] A Canadian mortality rate was then calculated for each of the five categories of rurality by combining all CSDs belonging to the same category. [28] Mortality rates among immigrant women were obtained through a national record linkage study of a large immigrant cohort (the detailed methods and results of these studies will be described in greater detail elsewhere in upcoming publications).

## Results

### Patterns of Mortality

#### Time Trends and Sex Ratios

In 1999, approximately 105,900 women died in Canada. The current five leading causes of death in women are heart disease, cancer, cerebrovascular disease, pulmonary diseases and pneumonia/influenza, which account for approximately 70% of all deaths. Mortality rates obviously increase dramatically with age, and this age effect is similar in both sexes (data not shown). A comprehensive description of these mortality patterns is available in a number of recent reports. [1,6]

**Figure 1:**
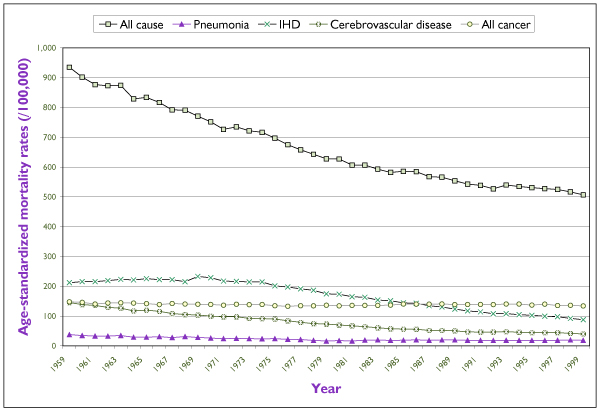
**Selected Cause-Specific Age-Standardized Mortality Rates, Women, Canada, 1959–1999. Age-standardized mortality rates (/100,000) **Source: Vital Statistics, Statistics Canada

**Figure 2:**
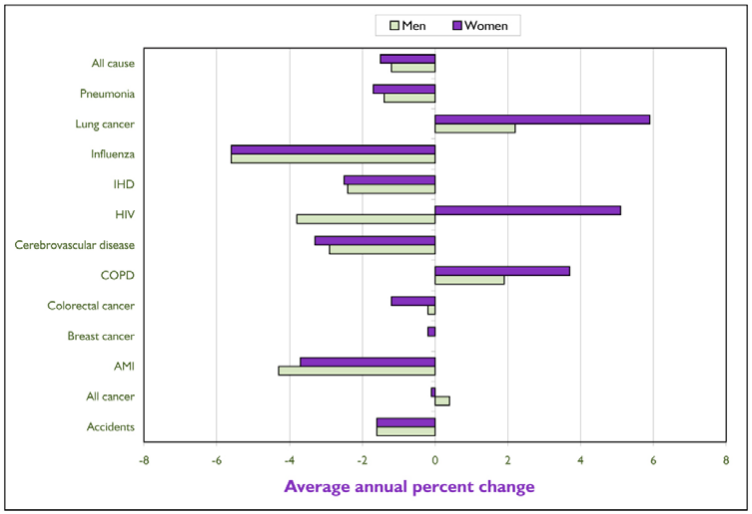
**Average Annual Percent Change in Age-Standardized Mortality Rate for Selected Causes, Canada, 1959–1999 **Source: Vital Statistics, Statistics Canada

Although mortality rates continue to be higher among men, they decreased significantly in both sexes between 1959 and 1999 (women had a slightly greater all-cause annual percentage decrease, 1.5%, as compared with 1.2% among men), as indicated in Figures 1 and 2. Younger age groups experienced a larger decrease in mortality (3.8 % and 3.9% among 0- to 19-year-old males and females respectively). These time trends varied by cause of death and sex: women had a greater average annual percentage decrease compared with men in deaths due to pneumonia, ischemic heart disease, cerebrovascular disease and colorectal cancer. Men had a greater average annual percentage decrease in deaths from influenza and acute myocardial infarction. For both sexes, death rates from lung cancer and chronic obstructive pulmonary disease (COPD) increased. Deaths from AIDS increased among women and decreased among men. Cancer was the only cause of death in which average annual mortality rates among men increased and those among females decreased.

**Figure 3:**
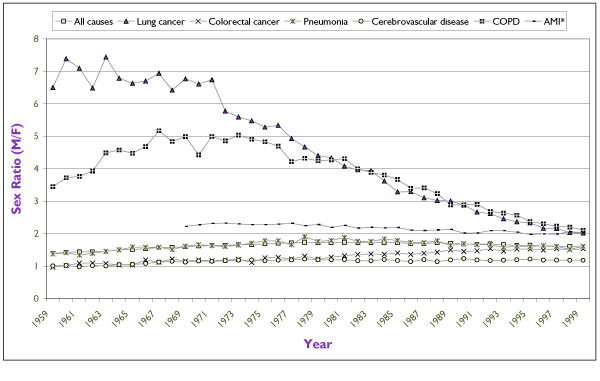
Age-Standardized Sex Ratios by Cause of Death, Canada, 1959–1999 *AMI rates start in 1969 Source: Vital Statistics, Statistics Canada

Mortality male-female sex ratios, presented in Figure 3, indicate that the sex gap in all-cause mortality increased in the 1960s and 1970s, and has been narrowing since the 1980s. This finding is mainly attributed to decreasing sex ratios for smoking-related causes such as lung cancer, acute myocardial infarction and COPD. However, sex ratios for colorectal cancer, cerebrovascular disease and pneumonia are increasing. Sex ratios were larger in the 20 to 44 age group (approximately 2:1) and more moderate in the 65+ age group (approximately 1.5:1) (data not shown).

#### "External" Causes of Death Preventable through Primary Prevention

**Figure 4:**
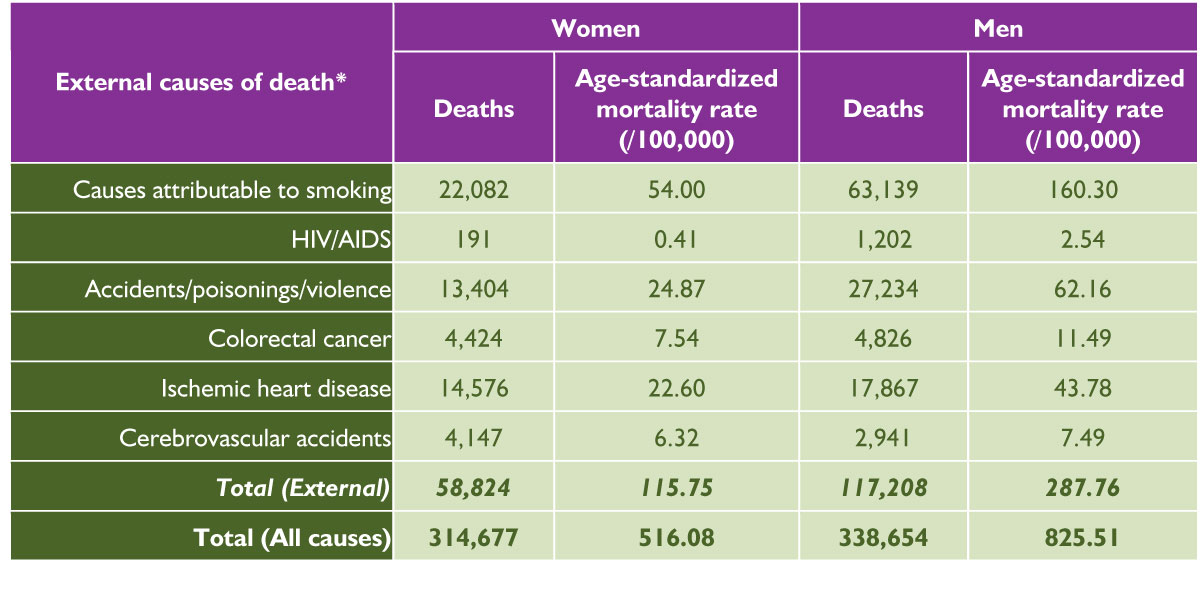
External Causes of Death, Canada, 1997–1999. *90% of HIV/AIDS cases, 40% colorectal cases, 25% IHD cases, 15% of CVA cases preventable through primary prevention Source: Vital Statistics, Statistics Canada

"External" causes of mortality included smoking-related deaths, HIV/AIDS, accidental deaths and a proportion of colorectal cancer preventable through primary prevention (physical activity and diet). Smoking-related causes of death include coronary heart disease, cerebrovascular disease, cancer and COPD. As indicated in Figure 4, a total of 55,824 deaths (115.75/100,000 or 19% of all deaths in women) among women were attributed to these preventable (through primary prevention) causes in 1997–1999, as compared with 117,208 (287.76/100,000 or 35% of all deaths in men) among men. Men had higher mortality rates for all these selected preventable causes, although the sex gap was most pronounced for HIV/AIDs.

#### Sex/Biology Related Specific Causes of Death

Some causes of death are biologically based, as they affect sex-specific organs and anatomy. These causes of death are referred to as "internal" causes of death and in women include conditions such as breast cancer, complications of pregnancy and gynecological cancers (more specifically, cancer of the ovary and cancer of the uterus); in men, internal causes include mainly cancer of the male genital organs, prostate diseases and excess perinatal mortality.

**Figure 5:**
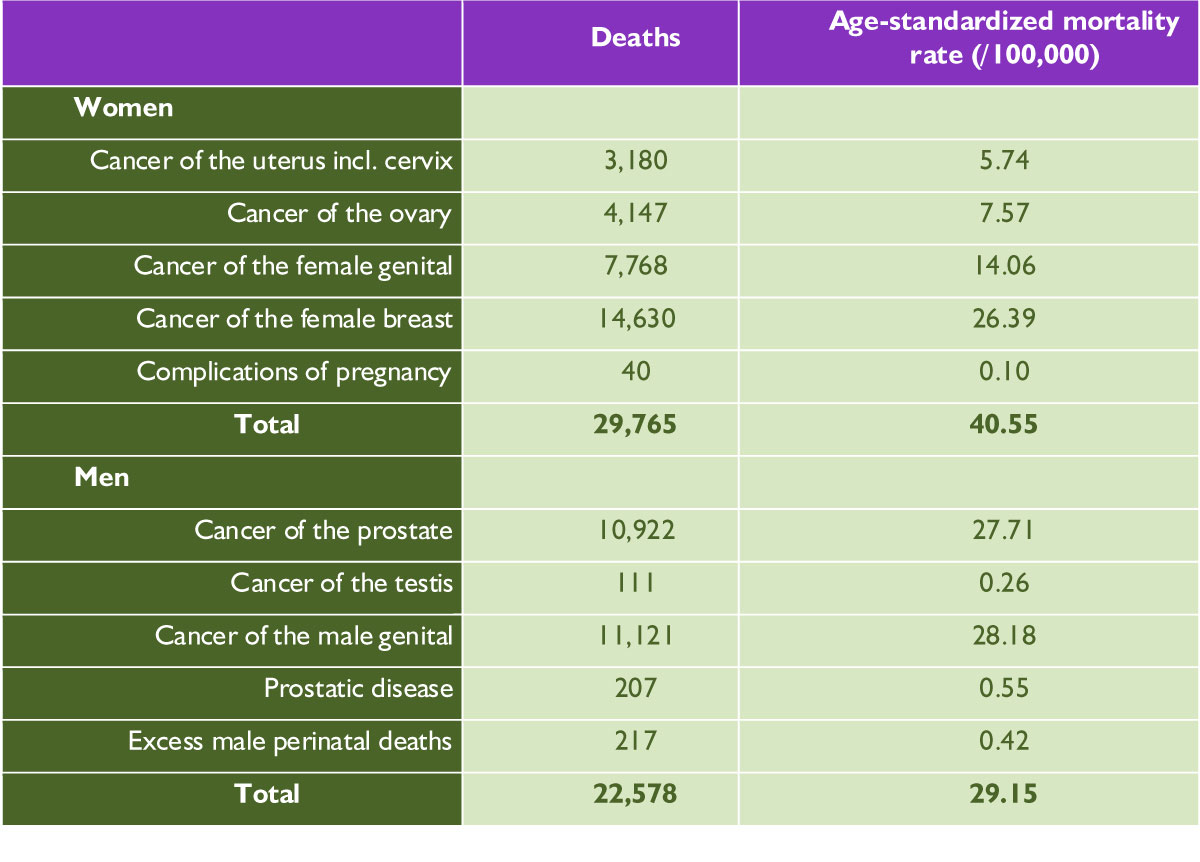
**Sex/Biology-Related Specific Causes of Death, Canada, 1997–1999 **Source: Vital Statistics, Statistics Canada

The relative significance of these biologically based causes of death for men and women are compared in order to assess the importance of biology with respect to mortality. The mortality rates are presented in Figure 5. Overall, internal causes of death are more common among women: 29,765 deaths (or 40.55/100,000) as compared with 22,578 (or 29.15/100,000) among men. A more detailed description of patterns of female cancers and pregnancy outcomes are presented in later chapters.

#### Amenable Causes

**Figure 6:**
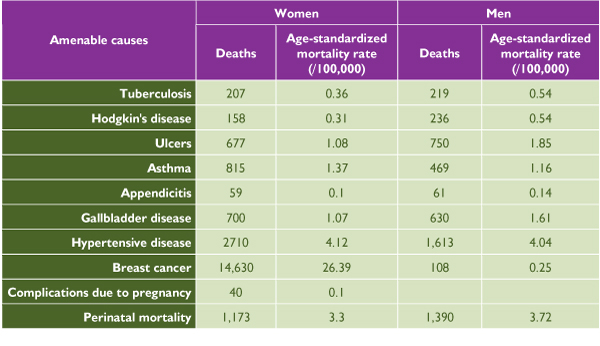
**Amenable Causes of Death, Canada, 1997–1999 **Source: Vital Statistics, Statistics Canada

Causes of death considered to be amenable to health care [19] include tuberculosis, hypertension, asthma, peptic ulcers, appendicitis and others, as listed in Figure 6. The overall mortality burden for these causes is higher among men than women, providing additional evidence that women may be at lower risk for some of these conditions and/or may use the health care system more appropriately than men. Hypertensive disease resulted in the highest mortality rates among both men and women. Asthma is the only amenable cause of death in which mortality rates are higher among women than men.

#### Life Expectancy

**Figure 7:**
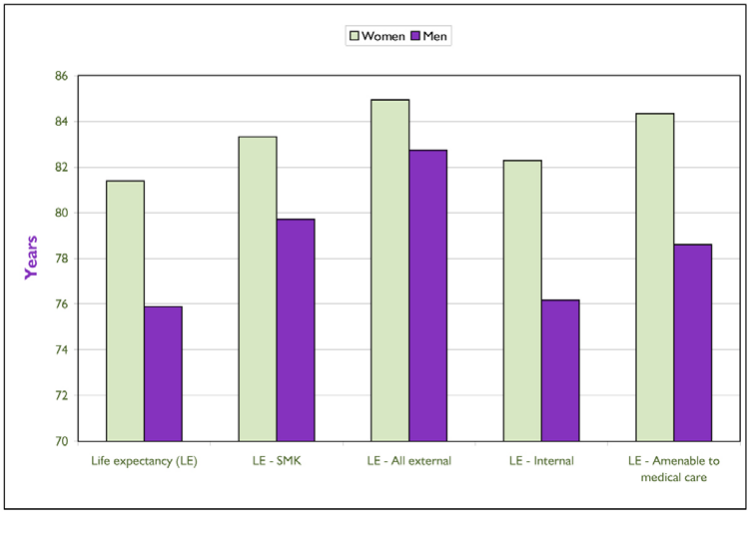
Life Expectancy at Birth of Canadian Women and Men, 1997–1999* *Smoking related: Coronary heart disease, cerebrovascular disease, lung cancer, cancer, COPD External non-smoking: HIV/AIDS, colorectal cancer, IHD, accidents, cardiovascular Internal: all female cancers, prostate cancer, pregnancy and complications, proportion of perinatal deaths that are higher in baby boys than girls Secondary prevention: TB, maternal, perinatal, Hodgkin's disease, asthma, hypertension, breast, IHD, appendicitis, gallbladder, peptic ulcers. Source: Vital Statistics, Census, Statistics Canada.

As indicated in Figure 7, life expectancy at birth is currently approximately 81.4 and 75.9 years for Canadian women and men respectively (a difference of 5.5 years). Smoking-related deaths (e.g. a proportion of lung cancer, cardiovascular disease, etc.) represent a major contributor to the gap between men's and women's life expectancies. By deleting these causes, it was estimated that if all Canadians were non-smokers, the life expectancy at birth would be approximately 83.3 and 79.7 respectively. Other external causes of death (the causes as described above) accounted for another significant portion of the difference between men and women's mortality and life expectancy.

All external causes of death combined (including smoking) were responsible for the majority of the difference in life expectancy between men and women. By excluding these causes preventable through primary prevention, the resulting life expectancy was 84.9 and 82.7 for women and men respectively (a difference of 2.2 years), indicating that women do not appear to have as large a biological survival advantage but, rather, are at lower risk of preventable deaths. In the hypothetical situation in which men's preventable mortality rates were the same as those of women, the life expectancy sex gap would be reduced to 1.65 years. This is further indicated by the following results: if sex-related causes of death (such as female cancers, prostate cancer, pregnancy complications, etc.) and mortality amenable to health care are excluded from the life expectancy calculations, the sex gap in life expectancy actually increases.

#### Potential Years of Life Lost and Premature Mortality

**Figure 8:**
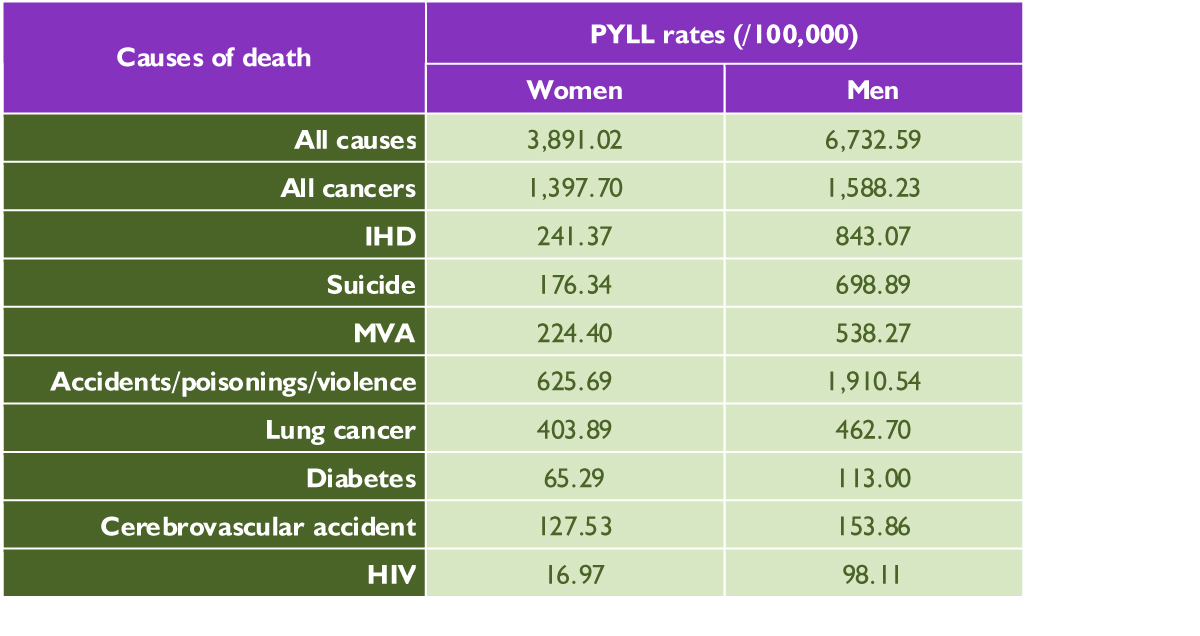
**PYLL Rates for Selected Causes of Death, Canada, 1997–1999 **Source: Vital Statistics, Statistics Canada

**Figure 9:**
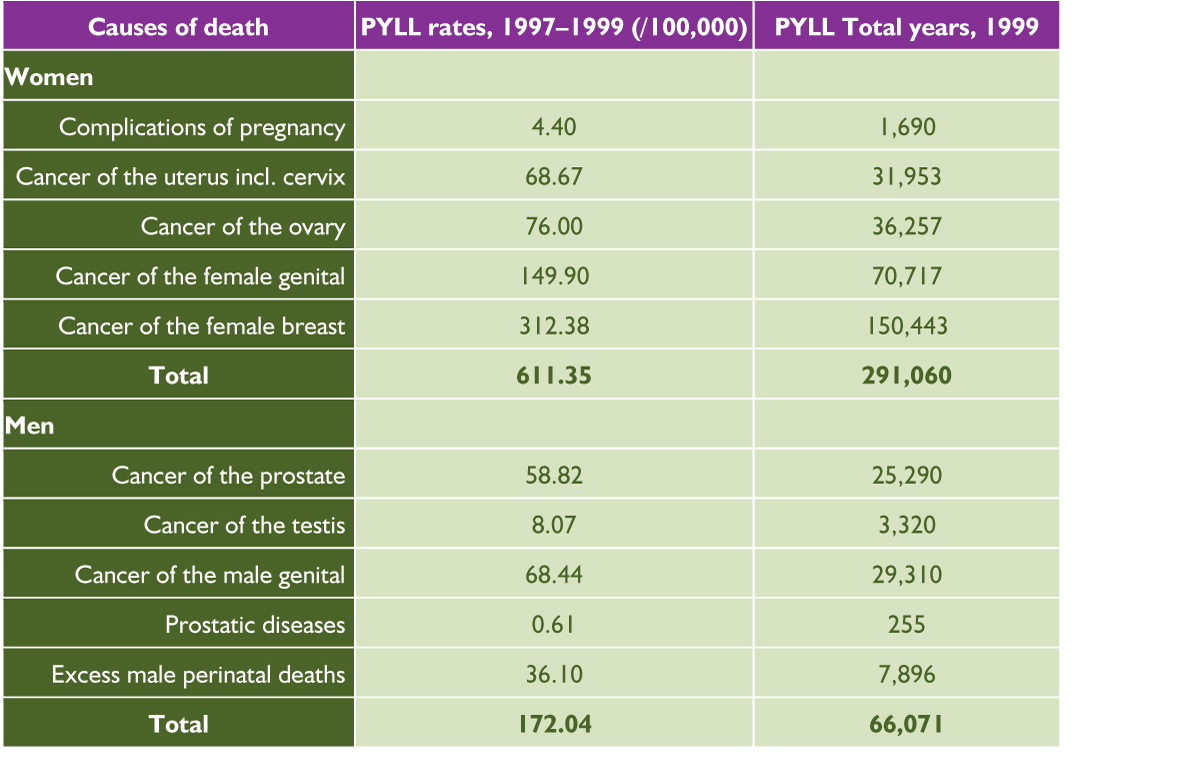
**YLL Rates and Totals for Sex-Specific Causes of Death, Canada **Source: Vital Statistics, Statistics Canada

Patterns of premature mortality among Canadian women and men, as expressed by potential years of life lost (PYLL), have been well described elsewhere. [1,6] Key differences by sex include the larger share of cancer as a cause of PYLL among women, and accidents represent a larger proportion of PYLL among men (Figure 8). The all-cause total PYLL rate per population in 1997–1999 was 3891/100,000 among women and 6733/100,000 among men, indicating a burden of premature death among men. However, as indicated in Figure 9, PYLL due to biology are much higher among women (approximately four times higher) than men. Interestingly, lung cancer has now surpassed breast cancer in terms of premature mortality among Canadian women.

### Health Expectancy

**Figure 10:**
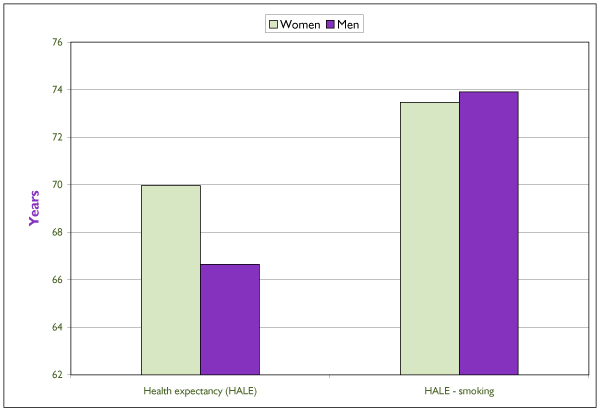
**Health Expectancy at Birth of Canadian Women and Men, 1997–1999 **Source: Vital Statistics, Statistics Canada

Health-adjusted life expectancy (HALE) at birth was 70.0 and 66.7 years for women and men respectively (1997–1999), a difference of 3.3 years. A reduction of the relative difference between men and women was observed when preventable causes such as smoking-related deaths and disability were excluded from HALE calculations (Figure 10). In fact, when smoking-related deaths were excluded, HALE actually became slightly lower for women (73.5 years) as compared with men (73.9 years).

### Subgroups of Canadian Women

Similar analyses of mortality and life expectancy by subgroups of women are somewhat limited by the variables available in current mortality databases in Canada. However, a number of comparisons are possible using area of residence information.

#### Provincial/Territorial Differences

**Figure 11:**
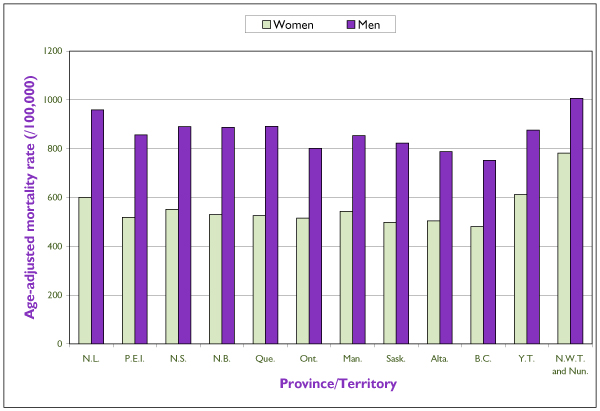
**Provincial/Territorial Differences in All-Cause, Age-Standardized Mortality Rates by Sex, 1997–999 **Source: Vital Statistics, Statistics Canada

The mortality patterns by province follow the same general patterns for women and men (increasing from west to east). However, the higher risk among Canadians living in the North is much more pronounced among women. In particular, the 1997–99 all-cause mortality rates among women living in the Northwest Territories was 60% higher than among women living in British Columbia, and 30% higher than women living in Newfoundland and Labrador. Among men, the corresponding differences ranged from 5% to 30% (Figure 11). Higher mortality rates were also observed among women living in the Yukon. These are new emerging patterns, as no such sex differences in the north-south gradient were observed in the early 1990s (data not shown).

#### Urban Versus Rural Area of Residence

**Figure 12:**
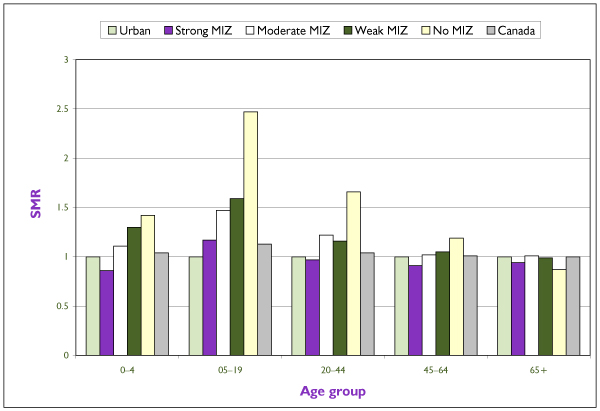
**All-Cause Standardized Mortality Ratio by Age and Rural/Urban Area of Residence, Women, Canada, 1986–1996 **All the SMR are statistically significant at p<0.05 except for Strong, Moderate and weak MIZ for 65+. Urban is the reference category, MIZ = metropolitan influence zone. Source: Vital Statistics, Census, Statistics Canada

Analyses of available information on province, and rural and urban area of residence, show important variations between subgroups of Canadian women, in particular significantly increased mortality rates among rural women as compared with their urban counterparts (which are approximately 250%, 160% and 20% higher among rural women aged 15 to 19, 20 to 44, and 45 to 64 respectively), as indicated in Figure 12. Causes of death contributing significantly to this rural/urban gradient include accidental deaths and chronic diseases such as diabetes and heart disease (data not shown). Similar rural/urban gaps in mortality were found for men.

#### Canadian Immigrant Women

**Figure 13:**
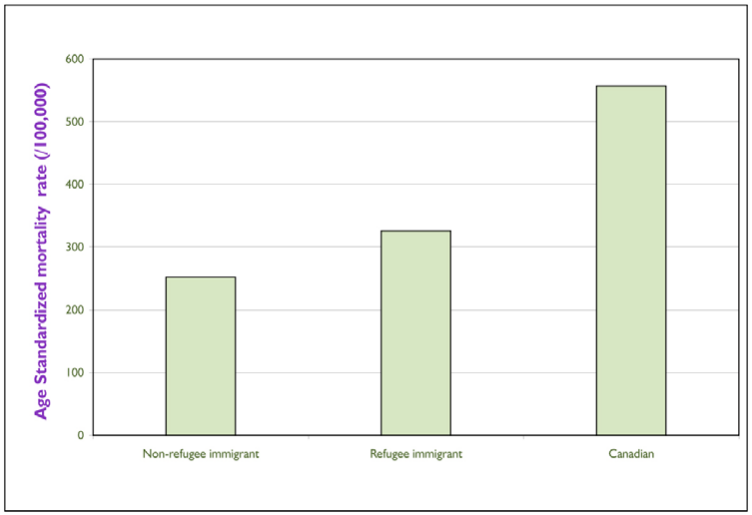
**Age Standardized Mortality Rates for Immigrant and All Canadian Women by Refugee Status, 1980–1999 **Source: Vital Statistics, Census, Statistics Canada

As indicated in Figure 13, Canadian immigrant women have a lower mortality rate than Canadian women overall. This provides further evidence of the well-known "healthy immigrant effect," a combination of the effect of self-selection and medical screening before immigration. However, important patterns are seen by cause of death and subgroup of immigrants (for example, between refugee and non-refugee women, and by country of origin), which indicate that some immigrant women are more vulnerable.

## Discussion

### Data Gaps and Recommendations

This chapter highlights key sex and gender patterns in mortality, and in life and health expectancy. In particular, although Canadian women tend to have lower mortality and longer life expectancy than men, this survival advantage does not appear to be biologically based, as most of the sex gap can be explained by higher mortality from preventable causes common to both sexes, such as smoking and accidents. This is further evidenced by the fact that men have a higher mortality rate from causes considered amenable to health care. Moreover, the mortality burden of biologically based, sex-specific causes (such as breast and prostate cancer) is actually greater among women in terms of mortality rates and potential years of life lost.

These findings are even more pronounced when a similar approach is used to examine years of healthy life (health adjusted life expectancy) experienced by Canadian women and men. In particular, it is clear that women have a shorter HALE when preventable causes of death are excluded. Although not calculated as part of the analyses in this chapter, it is expected that the relative disadvantage of women as compared with men in terms of cause-deleted HALE would be even more pronounced if preventable causes other than smoking-related deaths, such as injuries, were also deleted.

Given the higher prevalence of many conditions that cause significant disability but are currently less preventable (such as arthritis) among women, these results are of importance from a public health perspective. They provide additional insight into the nature of the sex gap in health and provide a useful tool to monitor changes in this health gap over time. A more detailed description of morbidity and disability among women may be found in the chapter entitled in "Morbidity Experiences and Disability among Canadian Women".

Significant disparities in mortality between different groups of Canadian women were found, including a larger mortality burden among rural and northern women. Although the rural/urban health gap was not found to be unique to women, women living in rural Canada have their own patterns of health outcomes and health determinants, such as lower employment rates and higher fertility rates than their urban counterparts. These findings provide further evidence of the unique health needs of rural women, which must be addressed through more targeted programs and policies. [29] There is a recognized and significant shortage of information on the health status and health outcomes of rural Canadians. Key findings presented in this chapter highlight the need for enhanced interventions for improving the health outcomes of rural women and for further research, particularly into the prevention of accidental deaths and access to care for chronic diseases in rural women. (More detailed analyses and results on rural/urban differences, as well as on the health of immigrant women, will be available and published as part of more in-depth individual articles.)

The findings on mortality and on life and health expectancy presented here provide further evidence supporting the need to develop more targeted interventions aimed at reducing the sex and gender inequalities in life and health expectancies. Clearly, the factors having the strongest impact on the sex gap are smoking and external causes of death such as accidents and suicide. As smoking-related deaths and disability continue to increase among women and decrease among men, this sex gap is expected to continue to narrow.

Information on mortality and on life and health expectancy by gender-related variables such as SES and women's roles is currently limited in Canada. New studies on mortality and social determinants of health will be greatly facilitated by enhanced data availability, such as the future national linkage of census and mortality data, and will provide increased capacity for gender analyses in this area. Knowledge of the unique mortality patterns by subgroups of women will be enhanced by a number of current national initiatives in the area of immigrant and rural health.

The measures currently available for HALE are mainly based on a limited scope of physical functioning. More research needs to be devoted to the development of more gender-sensitive measures of HALE to consider the much broader nature of the problems affecting women's health.

With the changing patterns of avoidable mortality (such as smoking-related deaths and deaths from HIV/AIDS), projections of life expectancy and mortality rates would be very useful in planning for gender- and sex-specific interventions aimed at reducing sex and gender disparities. More comprehensive analysis of the total burden of preventable causes of death in women and men and subgroups of women (e.g. due to breast cancer, heart disease, smoking) would provide insight for development of policies aimed at more vulnerable populations.

## Note

The views expressed in this report do not necessarily represent the views of the Canadian Population Health Initiative, the Canadian Institute for Health Information or Health Canada.
